# Editorial on Genetic Diversity of Plant Tolerance to Environmental Restraints

**DOI:** 10.3390/genes14111992

**Published:** 2023-10-25

**Authors:** Rudy Dolferus, Olive Onyemaobi

**Affiliations:** 1 Commonwealth Scientific & Industrial Research Organisation (CSIRO) Agriculture & Food, GPO Box 1700, Canberra, ACT 2601, Australia; rdolferus58@gmail.com; 2 CSIRO Agriculture and Food, Floreat, WA 6014, Australia

Environmental restraints like cold, drought and heat adversely affect growth and development in different ways and at different plant developmental stages, leading to reduced crop yield. The reduction in global crop production is also exacerbated by rapid changes in climate conditions that affect duration, timing of seasonal occurrence and severity of abiotic stress events. To address the threat of climate change and secure food supplies for a growing world population (estimated to reach 9.7 billion people by 2050), plant breeders are faced with the challenge of stabilising crop productivity through identification of key genetic traits for better adaptation to abiotic stresses and climatic extremes. Abiotic stress tolerance is controlled by complex gene networks. Although genetic variation in abiotic stress tolerance appears to be present in populations adapted to different climates, the complexity and redundancy of gene networks controlling abiotic stress tolerance has made it very difficult for scientists to come up with straightforward genetic solutions. QTL mapping for drought, heat and chilling tolerance has always led to the identification of multiple QTL with a low phenotypic contribution. This makes it impossible to reliably fine-map QTL for marker development. Another issue is the lack of reliable and objective phenotyping methods: what is the quintessential trait to be phenotyped for drought, heat or cold tolerance? This is directly related to our lack of understanding of how plants respond to environmental challenges. It is likely that plants need a combination of different traits to provide sufficient stress tolerance. These traits will need to be identified first before they are used in phenotyping, mapping and marker development. They will then need to be combined in a stepwise breeding process to produce stress-tolerant crops. During these steps, any negative effects on yield and quality traits will need to be evaluated and eliminated. 

The emergence during the last decade of various “-omics” technologies (genomics, transcriptomics, proteomics and metabolomics) has offered powerful new methods to study the responses of plants to abiotic stress conditions. These technologies allow researchers to dissect which genes and regulatory/metabolic processes differ in the stress responses of “tolerant” and “sensitive” varieties. The papers presented in this Special Issue illustrate how our approach towards unravelling abiotic stress responses in plants is changing.

In the paper presented by [1] a variety of physiological traits that govern adaptation to three stress conditions (drought, ultraviolet-B stress and nitrogen stress) were investigated in ten sweet potato cultivars (*Solanum tuberosum*). Various shoot and root morphological, physiological and gas-exchange traits were scored for their contribution to growth and improved productivity under these three stress conditions, and cultivars with superior performance were identified for inclusion in breeding programmes. 

Another study on sweet potato [2] used RNA sequencing to identify transcriptome differences between phosphite-tolerant (Phi) and phosphite-sensitive cultivars. This study revealed differentially expressed genes (DEGs) related to ribosome, plant hormone signal transduction, photosynthesis and plant–pathogen interaction that function differently under Phi stress. 

*Pennisetum glaucum* (Pearl millet) is known to have a high tolerance to several environmental stresses, especially heat stress. Huang et al. explored the transcriptional changes in pearl millet under short- to long-term heat stress to identify key molecular mechanisms and genes related to heat tolerance [3]. Their study revealed that genes involved in protein folding pathways, reactive oxygen species (ROS) protection and flavonoid biosynthesis play an important role in heat tolerance.

Weighted correlation network analysis (WGCNA) is a system biology method used to describe gene association patterns between different biological samples. Wang et al. used this methodology to identify core genes linked to heat stress in heat-tolerant and heat-sensitive rice seedlings (*Oryza sativa*) [4]. The results indicate that ribosome and protein processing in the endoplasmic reticulum, starch and sucrose metabolism, as well as biosynthesis of secondary metabolites, function differently in heat-tolerant and heat-sensitive rice varieties.

Gene expression profiling was also used by [5]. to explore differences in cold acclimation in diploid and autotetraploid cytotypes of the perennial alpine plant *Ranunculus kuepferi*. The results show that tetraploids have a better cold acclimation potential than diploids, enabling them to better adapt to colder habitats in the mountains. 

Transcriptome profiling was used by [6] to explore the effect of drought stress during the reproductive stage in wheat (*Triticum aestivum*). By comparing the response of drought-tolerant and drought-sensitive wheat varieties, it was revealed that the control of stomatal conductance differs remarkably between the varieties. The transcriptome profiles also show major differences in the expression of genes involved in hormone metabolism and signalling (auxin, ABA and ethylene). 

The reproductive stage in crop plants is generally highly sensitive to a variety of abiotic stresses. This was further illustrated by [7] who studied the role of source/sink relationships and sugar transport during male reproductive development in rice. Their paper provides an overview of genes involved in sugar translocation from source tissues to reproductive sink tissues and transcription factors that play a regulatory role in these processes.

A major stumbling block in improving abiotic stress tolerance is reliable phenotyping. This is particularly true for field phenotyping, where plants are often challenged by different stresses at the same time. Controlled environment experiments can be used to avoid the variability of field phenotyping, but in crop species (e.g., cereals), field performance under stress conditions is the ultimate litmus test to judge which varieties are truly stress tolerant. This Special Issue, therefore, also includes one paper to illustrate the challenges met when conducting field phenotyping of chilling and frost tolerance in wheat. The unreliability of chilling/frost conditions in the field due to day-to-day and season-to-season variation in weather conditions has necessitated Leske and Biddulph to look for new methods to conduct their field experiments by using automated diesel heaters to create a temperature contrast between field plots [8]. 

Although plant biologists can make use of the many powerful tools provided by the “omics” era, it will ultimately depend on progress made in understanding what is required for improving abiotic stress tolerance in plants and how to apply this knowledge for improving phenotyping methods that will ultimately lead to breakthroughs in abiotic stress tolerance in crop plants.
